# Characterization of *Alstrom Syndrome 1* (*ALMS1*) Transcript Variants in Hodgkin Lymphoma Cells

**DOI:** 10.1371/journal.pone.0170694

**Published:** 2017-01-30

**Authors:** Katarina Braune, Ines Volkmer, Martin S. Staege

**Affiliations:** Department of Pediatrics, Martin Luther University Halle-Wittenberg, Halle (Saale), Germany; European Institute of Oncology, ITALY

## Abstract

The Alstrom syndrome gene (ALMS1) is one of the largest disease associated genes identified today in the human genome and is implicated in cell cycle control, ciliogenesis, endosome recycling and intracellular transport mechanisms. ALMS1 mutations cause Alstrom syndrome, a rare genetic disorder. However, its function is not completely understood. DNA microarray analysis suggested that ALMS1 might be differentially expressed between Hodgkin lymphoma (HL) cells and normal tissues. By using reverse transcription-polymerase chain reaction (RT-PCR) we detected low but variable expression of ALMS1 in HL cell lines with highest expression in KM-H2 cells. Immunofluorescence indicated centrosomal accumulation of ALMS1 protein in HL cells. Knock-down of ALMS1 in KM-H2 cells had no impact on viability or cytotoxic drug sensitivity of these cells. Sequencing of RT-PCR products from HL cell lines identified three variable regions in ALMS1 transcripts that affect exons 2, 13, and 23. One of these variants was characterized by splicing out of exon 13. The other variants are characterized by two alternative 5 prime ends or alternative 3 prime ends. Structure prediction of the corresponding RNAs and proteins suggest that the different transcript variants might affect posttranscriptional regulation and ligand binding.

## Introduction

Hodgkin lymphoma (HL) is a lymphoproliferative disease of unknown etiology. The prognosis for HL patients steadily improved over the last few decades, particularly with the introduction of combined radio-chemotherapy [1]. With a five-year survival rate of over 90% in Europe and the United States [2,3], Hodgkin lymphoma today has one of the best prognoses among all childhood malignancies. Despite this development, not all patients can be cured with currently established therapy protocols. Furthermore, conventional cancer therapy is associated with numerous side effects and serious long-term complications such as secondary malignancies [4]. By understanding the molecular mechanisms of tumor development, progression and drug resistance, potential candidate genes can be identified as new targets for HL therapy.

In the past, DNA microarray analyses were used in order to identify HL specific expression profiles and thus potential candidate genes for HL therapy. As observed in previous studies [5,6], HL cell lines have differential gene expression patterns compared to hematopoietic cells and other normal body tissues. Some of these genes are involved in cell cycle regulation (*e*.*g*. *cyclin D2*), others are markers for hematopoietic cells (*e*.*g*. the *interleukin 21 receptor*). Gene expression profiles of the four HL cell lines L-1236 [7], L-540 [8], KM-H2 [9], and HDLM-2 [10] were shown to be rather similar. Therefore, a possible correlation to the pathogenesis of HL was suspected.

According to DNA microarray data, a probe set specific for the Alstrom syndrome gene (*ALMS1)* was among the probe sets with lowest signal intensities in HL cell lines compared to normal tissues [6]. *ALMS1*, (Gene ID: 7840) is located at position p13 of the short arm of human chromosome 2. The genomic reference sequence comprises 224,160 base pairs (bp). The mRNA reference sequence comprises 12,928 bp (both according to the National Center of Biotechnology Information (NCBI) database). Alternatively spliced transcripts encoding different isoforms of the ALMS1 protein have been described, however, the exon structure of individual cDNAs has not yet been conclusively identified [11,12]. The longest *ALMS1* transcript encodes a 461 kDa protein with 4,169 amino acids and contains a large tandem repeat domain with 34 incomplete repetitions of 45 to 50 amino acids, a leucine zipper and a polymorphic strand with 12 to 20 glutamic acid residues followed by 7 alanine residues near the N-terminus [12,13]. Some protein variants might have distinct intracellular localizations and may perform different functions [13,14]. The C-terminus shows similarities to the centrosome proteins C10orf90 (chromosome 10 open reading frame 90) and CEP295 (centrosomal protein 295), both also with unknown function. This region is called ALMS motif [11,15]. *ALMS1* is ubiquitously expressed in human tissues at low levels. Heart, placenta, skeletal muscle, pancreas and testis show higher expression levels [12]. The ALMS1 protein is found in the centrosomes and basal bodies of ciliated cells. Roles in microtubular organization, intracellular transport mechanisms and the normal function of cilia have been suggested [16,17]. *In vitro* knockdown of *ALMS1* in murine kidney cells using short interfering RNA led to abnormally stunted cilia [16], which supports a role for *ALMS1* in ciliary function and ciliogenesis. The transcription of *ALMS1* is regulated by the ubiquitous factor SP1 (specificity protein 1) and RFX1 (regulatory factor X1), which are already known to regulate genes involved in the function and maintenance of cilia [18]. Over 120 mutations in *ALMS1* are known to cause Alstrome syndrome (Online Mendelian Inheritance in Man accession number 203800), a rare multi-systemic disease with less than 1,000 reported cases world-wide [19]. The majority of these mutations are nonsense and frame-shift mutations, mainly located in exons 8, 10 and 16 that result in premature termination codons [11,12,20]. The ubiquitous expression of *ALMS1* is a possible explanation for the syndromic nature of the disease, the impairment of several organ systems and the variety of phenotypes in affected individuals [11]. First symptoms occur in early childhood as nystagmus and photophobia leading to progressive retinopathy and juvenile blindness, obesity, and dilated cardiomyopathy [14,20,21]. All Alstrom patients are affected by early-onset adiposity and an impaired glucose tolerance with hyperinsulinemia at an early stage of the disease [11]. A dysfunction at the neuronal level is assumed to be the cause of the sensory deficit and increased appetite [11]. Other clinical features of Alstrom syndrome usually occur in childhood or early adolescence and include sensorineural hearing loss, pulmonary fibrosis, hepatic and renal dysfunction [20,21]. The life expectancy of Alstrom patients is highly variable and rarely exceeds 40 years [20].

## Materials and Methods

### Ethics statement

This study uses only commercially available anonymous RNA sources.

### Cells and cell culture

HL cell lines L-1236, L-428, L-540, HDLM-2, and KM-H2 [7–10] were obtained from the Deutsche Sammlung von Mikroorganismen und Zellkulturen (DSMZ), Brunswick, Germany. All cells were cultured as cell suspension in RPMI-1640 medium with 10% fetal calf serum (Biochrom AG, Berlin, Germany) and 1% penicillin/streptomycin (PAA, Pasching, Austria) at 37°C in a humidified atmosphere with 5% CO_2_. Every 2 to 3 days the cells were split 1:3 into fresh medium.

### Generation of cDNA

RNA from testis was purchased from Agilent (Santa Clara, CA). RNA from HL cell lines was isolated using TRIzol^TM^ reagent (Invitrogen, Karlsruhe, Germany) following the manufacturer’s protocol. RNA was transcribed into cDNA using the following conditions: 1 μL oligo-dT_12-18_ primer or *ALMS1* specific primer 5’-TGG ACA AGT CAG TTG TTC TTC C-3’, 1 μL dNTP mix (10 mM), 1 μL RevertAid H Minus MuLV reverse transcriptase (Fermentas, St. Leon Roth, Germany), 4 μL 5x RT buffer, 2 μg RNA in a total volume of 20 μL. Reverse transcription was carried out at 37°C for 60 min. Thereafter, samples were incubated for 5 min at 90°C.

### Reverse transcription-polymerase chain reaction (RT-PCR)

After reverse transcription of 2 μg RNA into cDNA, polymerase chain reaction was performed using one of the following conditions: (a) 2.5 μL 10x PCR buffer, 1.5 μL MgCl_2_ (25 mM), 0.5 μL dNTP mix (10 mM), 0.25 μL sense primer (25 μM), 0.25 μL antisense primer (25 μM), 0.2 μL Taq polymerase, 17.8 μl H_2_O, 2 μL cDNA; (b) 5 μL GoTaq buffer, 0.5 μL dNTP mix (10 mM), 0.25 μL sense primer (25 μM), 0.25 μL antisense primer (25 μM), 0.2 μL Taq polymerase, 16.8 μL H_2_O, 2 μL cDNA. After initial denaturation at 94°C for 5 min, PCR was performed with 35 cycles (*actin beta (ACTB)*: 19 cycles): 94°C, 30 s; 60°C, 30 s; 72°C, 45–60 s.

Sequence specific primers were designed using Primer3plus software (http://www.primer3plus.com) [22]. Primer combinations are shown in Table 1. PCR products were subjected to 1.5% agarose gel electrophoresis in the presence of ethidium bromide.

**Table 1:** Primer combinations used in this study.

No.	Name	Primer sequence (5’-3’)	Target
1	ACTB_L, ACTB_R	GGCATCGTGATGGACTCCG, GCTGGAAGGTGGACAGCGA	*ACTB*
2	ALMS1_1AL, ALMS1_2R	ATAGACGACGAGGAGGACGA, AGAACCCCGCTGAGCTGT	*ALMS1*, exon 1–2
3	ALMS1_1L, ALMS1_3R	AGCGAGACACCAACATGGA. TCTGTTGGGTTCCTTTACTTGA	*ALMS1*, exon 1–3
4	ALMS1_1AL, ALMS1_4BR	ATAGACGACGAGGAGGACGA, TCAGAGGTGCAAAACTTAGT	*ALMS1*, exon 1–4
5	ALMS1_1L, ALMS1_4R	AGCGAGACACCAACATGGA, TCAGAGGTGCAAAACTTAGTTCA	*ALMS1*, exon 1–4
6	ALMS1_1AL, ALMS1_4AR	ATAGACGACGAGGAGGACGA, AGGCAAATCAGGAGAAGCAA	*ALMS1*, exon 1–4
7	ALMS1_1BL, ALMS1_4AR	TAGACGACGAGGAGGACGAG, AGGCAAATCAGGAGAAGCAA	*ALMS1*, exon 1–4
8	ALMS1_2L, ALMS1_2R	TGACCTGTCATGTATGGCAAC, AGAACCCCGCTGAGCTGT	*ALMS1*, exon 2
9	ALMS1_2L, ALMS1_3R	TGACCTGTCATGTATGGCAAC, TCTGTTGGGTTCCTTTACTTGA	*ALMS1*, exon 2–3
10	ALMS1_3L, ALMS1_4R	TCTTGGCATTGTCTTCCTCA, TCAGAGGTGCAAAACTTAGTTCA	*ALMS1*, exon 3–4
11	ALMS1_4L, ALMS1_8.0R	GCTTCTCCTGATTTGCCTTT, CCCCCTATGTGAGTGGGAAC	*ALMS1*, exon 4–8
12	ALMS1_8.0L, ALMS1_8.1R	TGTCCCTTGAGGACCTGTCT, AGTGAAGAGGACGCAGAGGA	*ALMS1*, exon 8
13	ALMS1_8.1L, ALMS1_8.2R	CTGGCCTACCAACAGTACCC, GAGCTGGAAGGGGAGGTTAC	*ALMS1*, exon 8
14	ALMS1_8.2L, ALMS1_8.2R	GGGACACCAACTCCAACC, GAGCTGGAAGGGGAGGTTAC	*ALMS1*, exon 8
15	ALMS1_8AL, ALMS1_8AR	TGACCAGACAACTGGCATGT, CAGCTGGTCCAGGAGTGG	*ALMS1*, exon 8
16	ALMS1_8AL, ALMS1_8BR	TGACCAGACAACTGGCATGT, CTAGGCTTCTCCCTCTGTG	*ALMS1*, exon 8
17	ALMS1_8AL, ALMS1_8DR	TGACCAGACAACTGGCATGT, TGTTGGTGTTGGGACAGTCT	*ALMS1*, exon 8
18	ALMS1_8.3L, ALMS1_8.4R	GGACCTTTAGGTTCCAGTGC, CCAGGAACCCCTATTGCTTT	*ALMS1*, exon 8
19	ALMS1_8.4L, ALMS1_8.5R	GCCGCATTTTACTGAAGCAG, CGTGGAAGAGGGTTCTTTGA	*ALMS1*, exon 8
20	ALMS1_8.6A_L, ALMS1_8.6A_R	CCTTGCCCGTTTCAGAGATA, AACATCAGAAACTGATTCCA	*ALMS1*, exon 8
21	ALMS1_8L, ALMS1_9R	CCTGCTCGTGGGACAGTAAT, TGGACAAGTCAGTTGTTCTTCC	*ALMS1*, exon 8–9
22	ALMS1_8.5L, ALMS1_9R	CTGAAGAAATCCAGGATGCAG, TGGACAAGTCAGTTGTTCTTCC	*ALMS1*, exon 8–9
23	ALMS1_8.6A_L, ALMS1_9R	CCTTGCCCGTTTCAGAGATA, TGGACAAGTCAGTTGTTCTTCC	*ALMS1*, exon 8–9
24	ALMS1_9L, ALMS1_10R	TTCAATTTAGCACATGATTGTGG, AGAAATGGGAGACGGAAGGT	*ALMS1*, exon 9–10
25	ALMS1_10L, ALMS1_12R	GTGCAGATGACCATGTGAGG, CTGAATGGGAGCTTTCAACG	*ALMS1*, exon 10–12
26	ALMS1_B5, ALMS1_A3	ACGCAAAGCTCCTGTCAAGT, GAGGTTCTCATCCCCAGTGA	*ALMS1*, exon 11–14
27	ALMS1_B5, ALMS1_16R	ACGCAAAGCTCCTGTCAAGT, AAACGATCAAGTCGGTCCAC	*ALMS1*, exon 11–16
28	ALMS1_A5, ALMS1_A3	ACTTCCCAGCTCAGGTGCTA, GAGGTTCTCATCCCCAGTGA	*ALMS1*, exon 13–14
29	ALMS1_16L, ALMS1_18R	TCCACAAAGGGATCAGAAGG, CAGGATAACCGGTAAAGAGCA	*ALMS1*, exon 16–18
30	ALMS1_16L, ALMS1_20R	TCCACAAAGGGATCAGAAGG, GCACATGCGATTCTGGTG	*ALMS1*, exon 16–20
31	ALMS1_18L, ALMS1_23R	ATTGTGAACGGTGCCAAAA, ATTGGACAAGGCCACTATGC	*ALMS1*, exon 18–23
32	ALMS1_19L, ALMS1_23R	TGTTCCTGTGGAAAATGTGG, ATTGGACAAGGCCACTATGC	*ALMS1*, exon 19–23
33	ALMS1_19L, ALMS1_3UTR	TGTTCCTGTGGAAAATGTGG, TGGGTTCTTTTCCCAGTGTC	*ALMS1*, exon 19–3’ UTR
34	ALMS1_23L, ALMS1_23R	CCCTGGGACTGACACAAGTT, ATTGGACAAGGCCACTATGC	*ALMS1*, exon 23
35	ALMS1_23L, ALMS1_3UTR	CCCTGGGACTGACACAAGTT, TGGGTTCTTTTCCCAGTGTC	*ALMS1*, exon 23–3’ UTR
36	ALMS1_DBL, ALMS1_DBR	TCTGGCTTGCACCCAGAATA, AAGAGGAGCCTAAGACATGC	*ALMS1*, (214707_x_at)
37	ALMS1_DL, ALMS1_DR	GGTGTGCAGATCACATGAGG, GCCTCCCAGGTTCAAGAGAT	Alu repeat, (214707_x_at)
38	ALMS1_UL, ALMS1_UR	TGTCCCTTGTCACATACAGCTT, CAATTTGAGGGAAATCTGCAA	*ALMS1 intronic transcript 1*
39	HPRT1_R, HPRT1_L	ACCAGTCAACAGGGGACATAA, CTTCGTGGGGTCCTTTTCACC	*HPRT1*

### Sequence analysis

For sequence analyses, PCR products were purified from agarose gels by using the MinElute Gel Extraction Kit (Qiagen, Hilden, Germany) or GeneJet Extraction Kit (Thermo Scientific, Waltham, USA) as described in manufacturer’s protocol. For sequencing, a 10 μl mix was used that contained 0.5 μL forward or reverse sequencing primers (25 μM), 4.0 μL BigDye Terminator Cycle Sequencing Kit reagent (Applied Biosystems, Foster City, CA, USA), 4.5 μL H_2_O and 1 μL DNA. Sequence analysis was performed by using an ABI Prism^TM^ 320 Genetic Analyzer (Applied Biosystems, Foster City, CA, USA). Sequences were further analysis with NCBI BLAST [23] (http://www.blast.ncbi.nlm.nih.gov/Blast.cgi) in order to align the obtained sequences with reference sequences.

### Quantitative real time polymerase chain reaction

Quantitative real time polymerase chain reaction (qRT-PCR) was performed as described using the Maxima^TM^ SYBR Green qPCR Master Mix (Fermentas, St Leon Roth, Germany) [6,24]. Relative expression values were calculated using the standard 2^-ΔΔCt^ method [25]. Transcripts were quantified in relation to the house keeping controls *actin beta (ACTB*) or *hypoxanthine phosphoribosyltransferase 1* (*HPRT1*).

### DNA microarray data

DNA microarray data from HL cell lines, HL biopsies, and normal tissues (Gene Expression Omnibus data sets GSE2109, GSE3526, GSE7307, GSE12427, GSE12453, GSE14879, GSE17920, GSE20011, GSE25986, and GSE26325 [6,26–32]) were used for characterization of *ALMS1* expression in varying cell types. From GSE3526 and GSE7307 a collection of normal tissue samples were selected. A complete list of the investigated Gene Expression Omnibus data sets is presented in S1 Table. Cel files were analyzed with Affymetrix Expression Console using Robust Multi-Array Analysis (RMA) algorithm.

### Knock-down of *ALSM1* in KM-H2 cells

Knock-down of *ALMS1* in KM-H2 cells was performed by using GeneSolution siRNAs Hs_ALMS1_5, Hs_ALMS1_6, Hs_ALMS1_7, and Hs_ALMS1_8 (Qiagen, Hilden, Germany). AllStars Negative Control siRNA (Qiagen) served as control. KM-H2 cells were transfected with 300 nM of siRNA by using the Amaxa Cell Line Nucleofector Kit T (Lonza, Basel Switzerland) according to manufacturer’s instructions. Knock-down of *ALMS1* was tested by qRT-PCR. In pilot experiments, strongest knock-down was observed with siRNA Hs_ALMS1_6 (target sequence AAG AAG TGA TGG ATA CTA CTA) which was, therefore, used for the subsequent experiments. Cisplatin sensitivity of HL cells was analyzed by propidium iodide staining and flow cytometry as described [6]. IC_50_ concentrations were calculated by using the IC_50_ Tool Kit (http://www.ic50.tk/).

### Intracellular detection of ALMS1 protein

ALMS1 protein was detected by immunofluorescence and flow cytometry essentially as described [33]. For this end, cells were fixed and permeabilized by using the cytofix/cytoperm kit (Becton-Dickinson, Heidelberg, Germany). Cells were incubated with anti-ALMS1 antibody ab84892 (abcam, Cambridge, U.K.). After extensive washing, cells were stained with fluorescein-labelled goat-anti-rabbit IgG (H+L) secondary antibody (ThermoFischer, Rockford, IL). Cells stained with secondary antibody alone served as control. Cells were analyzed by using an FACScan flow cytometer and CellQuest Pro software (Becton-Dickinson) as well as microscopically by using an Axiovert 25 microscope and AxioVision software version 3.1 (Zeiss, Jena, Germany). For fluorescence microscopy, cell nuclei and chromosomes were counterstained with Hoechst33258 (Sigma, St. Louis, MO). Micrographs were merged by using GIMP version 2.8.18 (https://www.gimp.org/) without further image enhancement.

## Results

**Fig 1:**
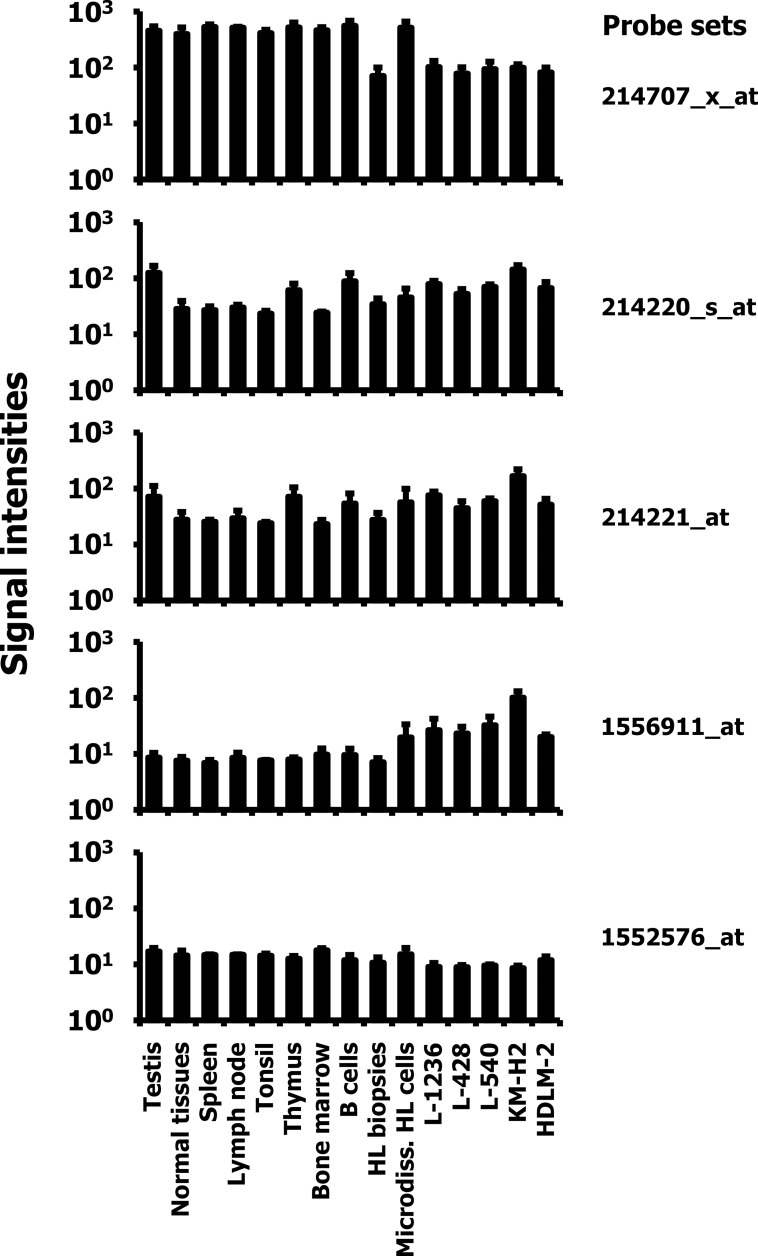
DNA microarray signal intensities of *ALMS1*, *ALMS1P*, and *ALMS1-IT1* specific probe sets. DNA microarray data from a panel of normal tissues, HL biopsies, micro-dissected HL cells, and HL cell lines [6,27–33] were analyzed for expression of *ALMS1* (probe sets 214707_x_at, 214220_s_at, and 214221_at), *ALMS1P* (probe set 1552576_at), and *ALMS1-IT1* (probe set 1556911_at). For normal tissues the expression in testis, B cells, diverse hematopoietic tissues and all other tissues are presented. The used data sets are summarized in S1 Table. Signal intensities of *ALMS1*, *ALMS1P*, and *ALMS1-IT1* related probe sets are presented as arithmetic means and standard deviations. Microdiss. HL cells: = HL cells that have been micro-dissected from HL biopsies.

Genes with differential expression in tumor cells compared to normal tissues may represent potential targets for cancer therapy. In our previous studies we identified a probe set (214707_x_at) annotated to *ALMS1* as differentially expressed between HL cell lines and a panel of normal tissues [6]. This probe set was one of 50 probe sets with very low signal intensities in HL cell lines. Using a larger number of publicly available microarray data sets from normal tissues, normal B cells, HL biopsies and HL cell lines, we reproduced this observation. As shown in Fig 1, the *ALMS1* probe set 214707_x_at showed very low signal intensities in HL cell lines in comparison to normal tissues including normal B cells. Interestingly, micro-dissected HL cells from biopsies showed higher signal intensities whereas non-dissected HL biopsies from a different study showed the same low signal intensities as established cell lines (Fig 1). The microarrays used in this study included 5 probe sets related to *ALMS1*. In addition to the probe set 214707_x_at, the arrays contained two *ALMS1* specific probe sets (214220_s_at and 214221_at), one probe set specific for the *ALMS1 pseudogene* (*ALMS1P; probe set 1552576_at*), and one probe set specific for the *ALMS1 intronic transcript 1 (ALMS1-IT1*; probe set 1556911_at). As shown in Fig 1, the signal intensities for these probe sets indicated no down-regulation in HL cell lines. Signal intensities for *ALMS1-IT1* were even higher in HL cell lines and micro-dissected HL cells than in normal tissues.

**Fig 2:**
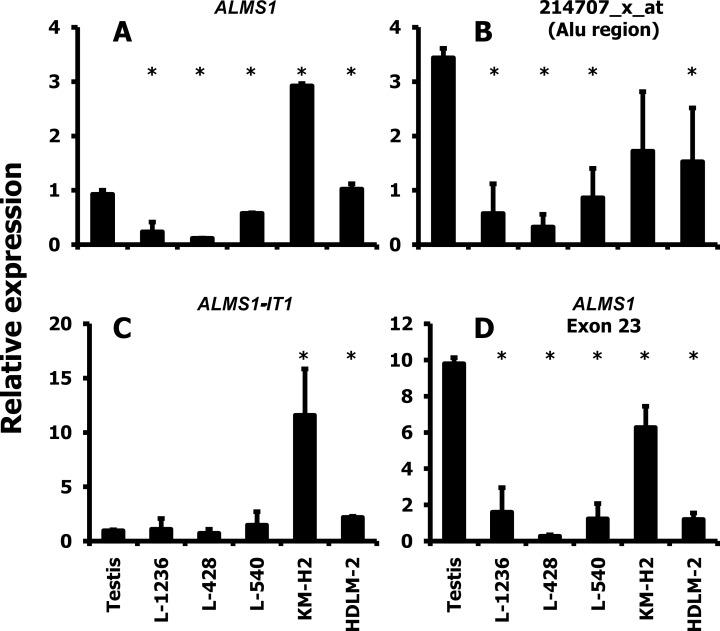
Relative expression level of ALMS1 and ALMS1-IT1 related transcripts in HL cell lines. Presented are results from qRT-PCR analyses with the indicated primer combinations. The following primer pairs were used: **A**) primer combination no. 36 from Table 1. This primer combination recognizes the target sequence of probe set 214707_x_at; **B**) primer combination no. 37. These primers are specific for the Alu sequence in the target sequence of probe set 214707_x_at; **C**) primer combination no. 38. These primers were designed for recognition of the target sequence form probe set 1556911_at (*ALSM1-IT1*); **D**) primer combination no. 34. These primers recognize the sequence from *ALMS1* exon 23. For each primer combination, the median of all samples was set as one and *ACTB* (primer combination no. 1) was used as housekeeping gene. Presented are means and standard deviations from 3–4 experiments. Asterisks indicate significant (p<0.05; Student’s t test) difference in comparison to testis.

Quantitative real-time PCR (qRT-PCR) was performed to further investigate *ALMS1* expression levels of HL cell lines together with testis as reference tissues as shown in Fig 2. We used 4 different primer pairs for detection of *ALMS1* and *ALMS1-IT1*. Taking together, PCR results indicate relatively low transcript levels of the 214707_x_at target sequence in most HL cell lines. Primer pair no. 36 (Fig 2A) was designed for recognition of the target sequence from probe set 214707_x_at. With this primer combination, the mean expression value of *ALMS1* was found to be higher in testis than in HL cells. The target sequence of probe set 214707_x_at includes an Alu repeat. Therefore, the virtually extreme down-regulation of *ALMS1* in the microarray data from HL cell lines might be due to cross hybridization with other Alu sequences (see below). We used a second primer pair that was designed for recognition of the Alu sequence in the 214707_x_at target sequence. This primer combination (no. 37 in Table 1) showed similar results as primer combination 36 (Fig 2B). Primers specific for the target sequence of probe set 1556911_at which corresponds to *ALMS1-IT1* indicated no down-regulation in HL cell lines (Fig 2C). Primers specific for exon 23 of *ALMS1* showed similar behavior as 214707_x_at specific primers (Fig 2D).

**Fig 3:**
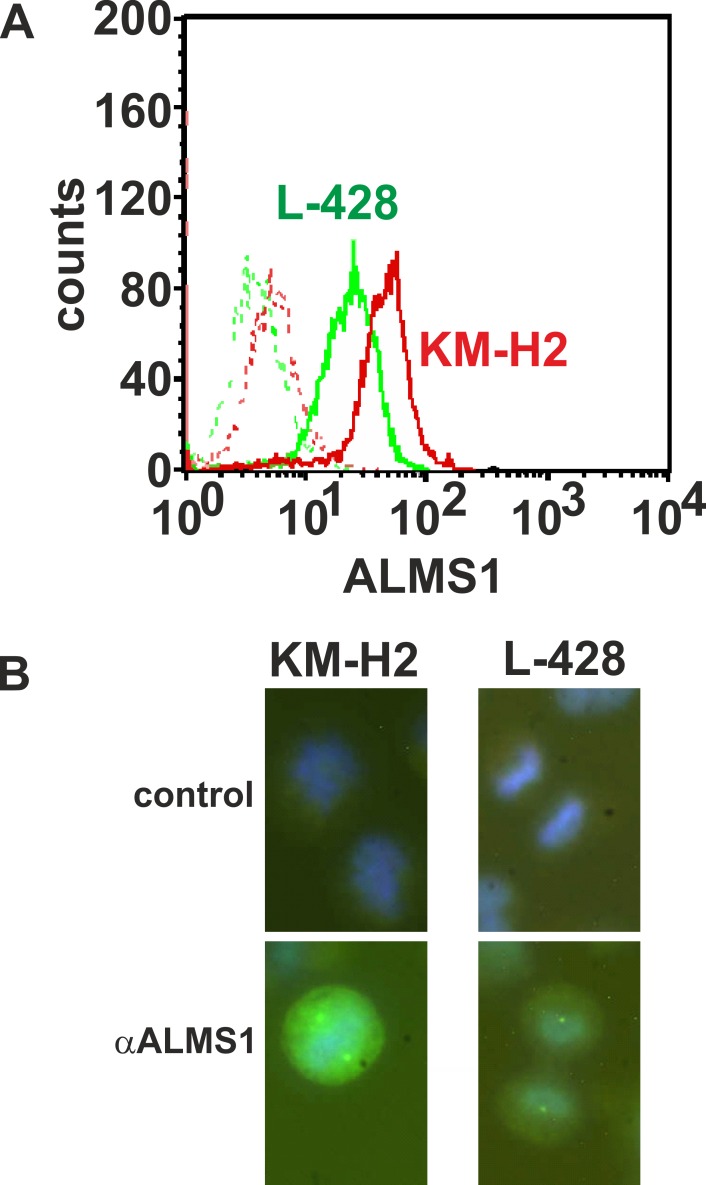
Detection and localization of ALMS1 protein in HL cells. **A)** Flow cytometric analysis of ALMS1 expression in HL cell lines. HL cell lines KM-H2 (red lines) and L-428 (green lines) were stained intracellularly with anti-ALMS1 antibodies (solid lines). Cells stained with secondary antibody alone served as control (dashed line). **B**) Fluorescence microscopic analysis of the cells from panel A. For staining of DNA, cells were counterstained with Hoechst33258. 40x objective, magnified.

We analyzed expression of ALMS1 in HL cell lines by using anti-ALMS1 antibodies. As expected from the qRT-PCR results, KM-H2 showed stronger ALMS1 staining than L-428 cells in flow cytometric analysis (Fig 3A). In both cells lines, a cytoplasmic staining with pronounced staining of centrosomes in dividing cells was detectable (Fig 3B).

**Fig 4:**
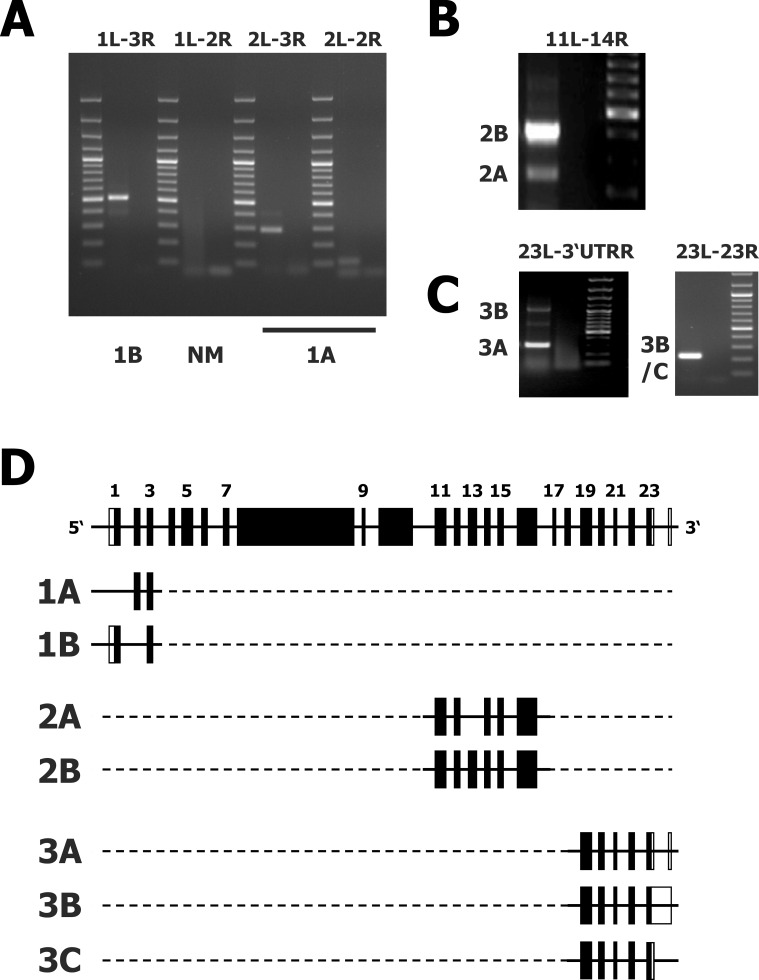
Transcript variants of *ALMS1* found in the L-1236 cell line. Presented are representative PCR results with primer combinations detecting varying exon combinations. For all primer combinations the left lane corresponds to the product from cell line L-1236 and the right lane corresponds to the no template control. **A)** Representative PCR result with primer combinations detecting exons 1–3 from *ALMS1*. The following combinations have been used (see Table 1): 1L-3R: combination no. 3; 1L-2R: combination no. 2; 2L-3R: combination no. 9; 2L-2R: combination no. 8. The PCR product (“1A”) corresponds to a transcript that includes exon 2 (and exon 3). The size of the PCR product “1B” corresponds to a transcript variant with missing exon 2. No product with the expected size of the reference sequence with exons 1, 2, and 3 was detectable. In addition, the primer combination 1L-2R should recognize the reference sequence with exon 1 and 2 (“NM”). No such PCR product could be detected. **B**) Representative PCR result with primer combination 25 from Table 1. The Two bands correspond to a transcript with missing exon 13 (“2B”) and a transcript variant that includes exon 13 (“2A”). **C**) Representative PCR result with primer combination 35 (left) and combination 34. Left panel: The bands “3A” and “3B” correspond to a transcript with truncated exon 23 and an additional exon in the 3’UTR region (“3A”) and a variant with complete exon 23 and complete 3’UTR including the additional exon (“3B”). Right: The used primer combination recognize exon 23 with the 3’UTR according to the reference sequence (“3C”) that is not present in variant “3A” but is included in variant “3B”. **D**) Schematic representation of the detected *ALMS1* transcript variants from L-1236 cells. Exons are presented by bars, introns by lines. The upper transcript corresponds to the complete NCBI reference sequence.

The PCR results with different primer combinations suggested that different regions of *ALMS1* might be expressed differentially in different cell types. The complete sequence of *ALMS1* transcripts in HL cells has not been determined. Therefore, sequence analyses were performed by sequencing the entire *ALMS1* cDNA of the cell line L-1236. As originally described [11,12], *ALMS1* is comprised of 23 exons. We designed primers for all of these exons. In addition, we designed primers for the 3’ untranslated region (3’UTR) of *ALMS1*. These primers were used because the public data bases include few nucleotide sequences (e.g. BE762689 and BE762726 [34]) which are obviously derived from *ALMS1* and contain an additional exon from the 3’UTR. Expression of all previously described exons was detected in L-1236 cells. By using different primer combinations (see Table 1) we discovered interesting *ALMS1* transcript variants as shown in Fig 4. These variants include (i) transcripts with or without exon 13, (ii) transcript variants with exon 1 or with exon 2 (but no variants with exons 1 AND 2), (iii) transcripts with alternative 3’ termini. The new variants have been submitted to GenBank (accession numbers EF151141, KX384043, and KX819248). As previously described [11], there is evidence of a splice variant lacking exon 2 and exon 2 is rarely found in human tissues. As our sequence analyses did not reveal any transcripts containing exon 1 and 2 in consecutive order, transcripts starting in exon two might be transcribed by an alternative promoter. The transcripts with alternative 3’ ends include a transcript with truncated exon 23 and an additional exon in the 3’ untranslated region.

## Discussion

Although the *ALMS1* gene is one of the largest disease-associated genes identified today in the human genome, its physiological function and pathological significance both for the etiology of Alstrom syndrome and carcinogenesis remains largely unknown.

qRT-PCR results confirmed our DNA microarray findings. Both methods revealed that the expression of transcripts corresponding to the *ALMS1* probe set 214707_x_at is reduced in HL cells compared to normal tissues. *ALMS1* has previously been discussed as a pro-apoptotic gene [35], because the lack of ALMS1 protein in fibroblasts results in over-expression of extracellular matrix components, a delayed cell cycle and resistance to apoptotic stimuli. We were unable to find an influence of *ALMS1* on viability or cytotoxic drug response of HL cells. After knock-down of *ALMS1* in cell line KM-H2 the cells showed the same viability and response to cisplatin as the control cells (S1 Fig). Other candidate genes might be more relevant for viability and chemo-resistance. For example, chemo-resistant L-1236 cells and L-428 cell harbor mutations in the tumor suppressor gene *TP53* whereas L-540 cells have an apparently wild-type *TP53* [36].

In DNA microarray analysis as well as in PCR analysis, KM-H2 cells demonstrated relatively high *ALMS1* expression compared to other HL cell lines. This might indicate relaxed control of *ALMS1* expression in this cell line. Taking into account the high expression of *ALMS1* in testis, it is interesting to note that DNA microarray data sets from KM-H2 cells show high signal intensities for several other testis specific transcripts. An example is shown in S2 Fig.

Signal intensities of probe set 214707_x_at showed high variability among data sets from different studies. We noticed that data sets with low 214707_x_at signal intensities also had low signal intensities for Affymetrix control probe sets (see S3 Fig), suggesting that differences in the experimental procedures used in different labs might influence signal intensities for this probe set. The Alu repeat in the target sequence of this probeset might facilitate such effects [37].

Our characterization of *ALMS1* transcripts indicated no mutations in the model cell line L-1236. Therefore the genomic structure of *ALMS1* appears intact in L-1236 cells. This is in agreement with published whole exome sequencing data from this cell line that indicate no *ALMS1* mutations [38].

**Fig 5:**
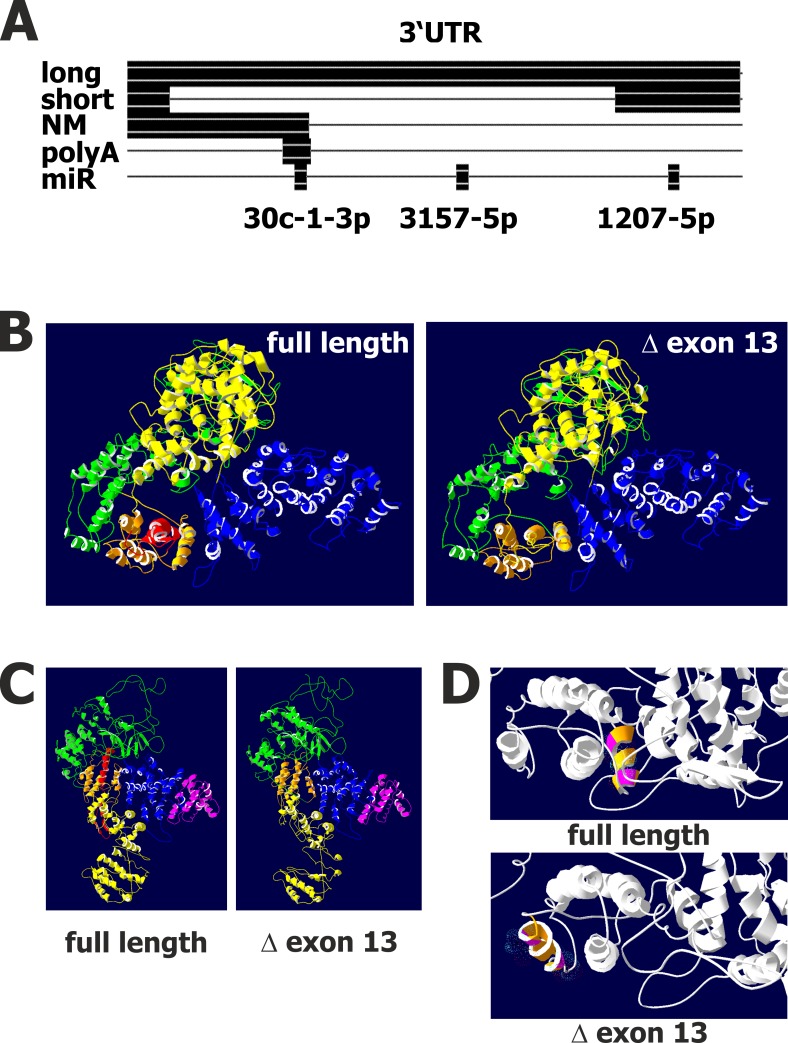
Putative functional consequences of *ALMS1* transcript variants. **A**) Schematic representation of predicted microRNA target sites and polyadenylation sites in transcript variants with different 3’UTR. Presented is an alignment of the 3’ ends of transcript variant 3A (short), transcript variant 3B (long) as well as the reference sequence (NM) and the predicted polyadenylation signal (polyA) and microRNA target sites (mIR). **B**) Predicted structures of the C terminal 1,500 amino acids encoded by the reference nucleotide sequence from human *ALMS1* (left structure) or the 1,443 amino acid encoded by the splice variant with lacking exon 13 (right structure). Individual globular domains are highlighted by different colors (from N terminus to C terminus: green, orange, yellow, blue). The amino acids encoded by exon 13 are colored red. **C**) The same structures as in panel B were presented after rotation. The ALMS1 homologue domain is highlighted in pink color. **D**) Detail of the predicted structures. The helix including the predicted Ca binding site in the splice variant with missing exon 13 is highlighted by coloring. This helix is exposed in the splice variant but not in the predicted structure of the full length protein.

The transcript variants identified in this study might have different functionalities (Fig 5). The two variants with different 3’ termini differ in the number of potential binding sites for microRNAs. Using the RegRNA2.0 tool [39] we predicted binding sites for microRNAs hsa-miR-30c-1-3p, hsa-miR-1207-5p, and hsa-miR-3157-5p in the 3’UTR from the longer transcript variant. Only one binding site for hsa-miR-1207-5p was predicted in the transcript variant with truncated exon 23 (Fig 5A). The Reference sequence NM_015120 contains only the hsa-miR-30c-1-3p binding site. The predicted polyadenylation signal is lost in the variant with truncated exon 23. The last exon is non-coding suggesting that this variant might be a target for nonsense mediated decay. The transcript variants affecting exons two or 13 encode different protein sequences. Exon two encodes for exactly 42 amino acids, exon 13 for exactly 57 amino acids. Therefore, transcript variants with lacking exon two or 13 result in deletion of these amino acids. We were unable to detect variants that include both exon one and two. Analysis of the intron between exon one and exon two with the Neural Network Promoter Prediction tool [40] suggests that an alternative promoter might be present upstream of exon two. In this case, the first possible open reading frame will start in exon three. Analysis with translation start site prediction tools [41,42] indicated that the corresponding ATG codon in exon 3 can serve as start codon (NetStart 1.0 [41] prediction scores for exon 1: 0.890, score for exon 3: 0.518; ATG^pr^ [42] reliability for exon 1: 0.60, for exon 3: 0.13). Alternatively, an additional exon upstream of exon one might exist which can provide a splice donor site. Until now, we were not able to identify such an additional exon. Exon 13 codes for 57 amino acids. Structure prediction with I-TASSER [43–46] suggests that the amino acids encoded by exon 13 are part of a globular domain and can be deleted without affecting the overall structure of the protein (Fig 5B). The ALMS motif is not affected by the splice variant (Fig 5C). Interestingly, the protein variant encoded by transcripts with missing exon 13 has a similar predicted topology like the variant including exon 13, but the individual helices in this domain change the position relative to the rest of the molecule (Fig 5D). The variant encoded by transcripts with missing exon 13 has a putative calcium binding site whereas this site is not accessible in the full-length variant of the protein (Fig 5D).

Taking together, we identified different *ALMS1* transcripts which encode different protein isoforms. Our study uses only established HL cell lines that recapitulate some but not all of the features of the lymphoma *in vivo*. Further studies are required to investigate the expression of *ALMS1* not only at the RNA but also at the protein level in HL patients. The very large open reading frame of *ALMS1* aggravates successful transgenic up-regulation of *ALMS1*. However, the physiological or pathophysiological functions of *ALMS1* transcripts and protein variants should be elucidated.

## Supporting Information

S1 Table Gene Expression Omnibus data sets used for analysis of *ALMS1* related transcripts. (PDF)

S1 Fig Knock-down of *ALMS1* has no impact on cisplatin sensitivity of KM-H2 cells. *ALMS1* specific siRNA was used for knock-down of *ALMS1* in HL cell line KM-H2. Presented are means and standard deviations from 3 independent transfection experiments (n = 4, control si RNA; n = 6, ALMS1 siRNA) **A**) *ALMS1* knock-down was validated by qRT-PCR using primer combination 28. The median of all samples was set as one and *HPRT1* (primer combination 39) was used as housekeeping control. Statistical significance was tested by Student’s t test. **B)** Cisplatin sensitivity was analyzed after transfection of KM-H2 cells with siRNAs. Presented are percentages of viable propidium iodide negative cells. **C)** 50%-inhibitory concentrations (IC50) for cisplatin treated KM-H2 cells after transfection were calculated by using the IC_50_ Tool Kit (http://www.ic50.tk/).
(TIF)

S2 Fig High signal intensities of a cancer testis antigen-specific probe set in KM-H2 cells. Presented are signal intensities from the microarray data sets summarized in S1 Table. Cel files were downloaded from the Gene Expression Omnibus data base and analyzed with Affymetrix Expression Console using Robust Multi-Array Analysis (RMA) algorithm. Presented are signal intensities from probe set 206626_x_at that is specific for synovial sarcoma X family member 1 (SSX1). Microdiss. HL cells: = HL cells that have been micro-dissected from HL biopsies.
(TIF)

S3 Fig Correlation between signal intensities from *ALMS1* probe set 214707_x_at and Affymetrix control probe sets. Presented are signal intensities from the microarray data sets summarized in S1 Table. Cel files were downloaded from the Gene expression Omnibus data base and analyzed with Affymetrix Expression Console using Robust Multi-Array Analysis (RMA) algorithm. Presented are signal intensities from probe set 214707_x_at in correlation to the indicated control probe sets.
(TIF)
