# Supplementary material for: Characterization of Alstrom Syndrome 1 (ALMS1) Transcript Variants in Hodgkin Lymphoma Cells
Source: PLoS One. 2017 Jan 30;12(1):e0170694. doi: 10.1371/journal.pone.0170694 (PMC5279758; doi:10.1371/journal.pone.0170694)
Supplement: S1 Table — (PDF) [file pone.0170694.s001.pdf]

Gene Expression Omnibus data sets used for analysis of ALMS1 related transcripts.

**Data set**	**tissue**
GSM175849	Accumbens
GSM175851	Accumbens
GSM175834	Adipose Tissue Omental
GSM175836	Adipose Tissue Omental
GSM80589	Adipose Tissue Subcutaneous
GSM80590	Adipose Tissue Subcutaneous
GSM80606	Adrenal Gland Cortex
GSM80607	Adrenal Gland Cortex
GSM175842	Amygdala
GSM175844	Amygdala
GSM176263	Aorta
GSM176264	Aorta
GSM175974	Bone Marrow
GSM176300	Bone Marrow
GSM175792	Breast
GSM175795	Breast
GSM80578	Bronchus
GSM80579	Bronchus
GSM175852	Cerebellum
GSM176157	Cerebellum
GSM80651	Cerebral Cortex
GSM80652	Cerebral Cortex
GSM175833	Cervix
GSM176130	Cervix
GSM175983	Colon Cecum
GSM175984	Colon Cecum
GSM175820	Coronary Artery
GSM175821	Coronary Artery
GSM175855	Corpus Callosum
GSM175857	Corpus Callosum
GSM176301	Deltoid Muscle
GSM176312	Deltoid Muscle
GSM175825	Dorsal Root Ganglia
GSM175827	Dorsal Root Ganglia
GSM80672	Endometrium
GSM80673	Endometrium
GSM80695	Esophagus

GSM80696	Esophagus
GSM176239	Fallopian Tube
GSM175859	Frontal Lobe
GSM175860	Frontal Lobe
GSM176447	Gloubus Pallidum External
GSM176448	Gloubus Pallidum External
GSM176436	Gloubus Pallidum Internal
GSM176445	Gloubus Pallidum Internal
GSM175814	Heart Atrium
GSM175815	Heart Atrium
GSM175817	Heart Ventricle
GSM175819	Heart Ventricle
GSM175861	Hippocampus
GSM175987	Hippocampus
GSM353923	HL biopsy
GSM447610	HL biopsy
GSM447611	HL biopsy
GSM447612	HL biopsy
GSM447613	HL biopsy
GSM447614	HL biopsy
GSM447615	HL biopsy
GSM447616	HL biopsy
GSM447617	HL biopsy
GSM447618	HL biopsy
GSM447619	HL biopsy
GSM447620	HL biopsy
GSM447621	HL biopsy
GSM447622	HL biopsy
GSM447623	HL biopsy
GSM447624	HL biopsy
GSM447625	HL biopsy
GSM447626	HL biopsy
GSM447627	HL biopsy
GSM447628	HL biopsy
GSM447629	HL biopsy
GSM447630	HL biopsy
GSM447631	HL biopsy
GSM447632	HL biopsy
GSM447633	HL biopsy
GSM447634	HL biopsy

GSM447635	HL biopsy
GSM447636	HL biopsy
GSM447637	HL biopsy
GSM447638	HL biopsy
GSM447639	HL biopsy
GSM447640	HL biopsy
GSM447641	HL biopsy
GSM447642	HL biopsy
GSM447643	HL biopsy
GSM447644	HL biopsy
GSM447645	HL biopsy
GSM447646	HL biopsy
GSM447647	HL biopsy
GSM447648	HL biopsy
GSM447649	HL biopsy
GSM447650	HL biopsy
GSM447651	HL biopsy
GSM447652	HL biopsy
GSM447653	HL biopsy
GSM447654	HL biopsy
GSM447655	HL biopsy
GSM447656	HL biopsy
GSM447657	HL biopsy
GSM447658	HL biopsy
GSM447659	HL biopsy
GSM447660	HL biopsy
GSM447661	HL biopsy
GSM447662	HL biopsy
GSM447663	HL biopsy
GSM447664	HL biopsy
GSM447665	HL biopsy
GSM447666	HL biopsy
GSM447667	HL biopsy
GSM447668	HL biopsy
GSM447669	HL biopsy
GSM447670	HL biopsy
GSM447671	HL biopsy
GSM447672	HL biopsy
GSM447673	HL biopsy
GSM447674	HL biopsy

GSM447675	HL biopsy
GSM447676	HL biopsy
GSM447677	HL biopsy
GSM447678	HL biopsy
GSM447679	HL biopsy
GSM447680	HL biopsy
GSM447681	HL biopsy
GSM447682	HL biopsy
GSM447683	HL biopsy
GSM447684	HL biopsy
GSM447685	HL biopsy
GSM447686	HL biopsy
GSM447687	HL biopsy
GSM447688	HL biopsy
GSM447689	HL biopsy
GSM447690	HL biopsy
GSM447691	HL biopsy
GSM447692	HL biopsy
GSM447693	HL biopsy
GSM447694	HL biopsy
GSM447695	HL biopsy
GSM447696	HL biopsy
GSM447697	HL biopsy
GSM447698	HL biopsy
GSM447699	HL biopsy
GSM447700	HL biopsy
GSM447701	HL biopsy
GSM447702	HL biopsy
GSM447703	HL biopsy
GSM447704	HL biopsy
GSM447705	HL biopsy
GSM447706	HL biopsy
GSM447707	HL biopsy
GSM447708	HL biopsy
GSM447709	HL biopsy
GSM447710	HL biopsy
GSM447711	HL biopsy
GSM447712	HL biopsy
GSM447713	HL biopsy
GSM447714	HL biopsy

GSM447715	HL biopsy
GSM447716	HL biopsy
GSM447717	HL biopsy
GSM447718	HL biopsy
GSM447719	HL biopsy
GSM447720	HL biopsy
GSM447721	HL biopsy
GSM447722	HL biopsy
GSM447723	HL biopsy
GSM447724	HL biopsy
GSM447725	HL biopsy
GSM447726	HL biopsy
GSM447727	HL biopsy
GSM447728	HL biopsy
GSM447729	HL biopsy
GSM447730	HL biopsy
GSM447731	HL biopsy
GSM447732	HL biopsy
GSM447733	HL biopsy
GSM447734	HL biopsy
GSM447735	HL biopsy
GSM447736	HL biopsy
GSM447737	HL biopsy
GSM447738	HL biopsy
GSM447739	HL biopsy
GSM312811	HL biopsy (microdissected)
GSM312812	HL biopsy (microdissected)
GSM312813	HL biopsy (microdissected)
GSM312814	HL biopsy (microdissected)
GSM312815	HL biopsy (microdissected)
GSM312816	HL biopsy (microdissected)
GSM312817	HL biopsy (microdissected)
GSM312818	HL biopsy (microdissected)
GSM312819	HL biopsy (microdissected)
GSM312820	HL biopsy (microdissected)
GSM312821	HL biopsy (microdissected)
GSM312822	HL biopsy (microdissected)
GSM371754	HL biopsy (microdissected)
GSM371755	HL biopsy (microdissected)
GSM371777	HL biopsy (microdissected)

GSM371780	HL biopsy (microdissected)
GSM956644	HL biopsy (microdissected)
GSM956645	HL biopsy (microdissected)
GSM956646	HL biopsy (microdissected)
GSM956647	HL biopsy (microdissected)
GSM956648	HL biopsy (microdissected)
GSM956649	HL biopsy (microdissected)
GSM956650	HL biopsy (microdissected)
GSM956651	HL biopsy (microdissected)
GSM956652	HL biopsy (microdissected)
GSM956653	HL biopsy (microdissected)
GSM956654	HL biopsy (microdissected)
GSM956655	HL biopsy (microdissected)
GSM956656	HL biopsy (microdissected)
GSM956657	HL biopsy (microdissected)
GSM956658	HL biopsy (microdissected)
GSM956659	HL biopsy (microdissected)
GSM956660	HL biopsy (microdissected)
GSM956661	HL biopsy (microdissected)
GSM956662	HL biopsy (microdissected)
GSM956663	HL biopsy (microdissected)
GSM956664	HL biopsy (microdissected)
GSM956665	HL biopsy (microdissected)
GSM956666	HL biopsy (microdissected)
GSM956667	HL biopsy (microdissected)
GSM956668	HL biopsy (microdissected)
GSM956669	HL biopsy (microdissected)
GSM956670	HL biopsy (microdissected)
GSM956671	HL biopsy (microdissected)
GSM956672	HL biopsy (microdissected)
GSM499724	HL Cell Line HDLM-2
GSM499732	HL Cell Line HDLM-2
GSM637963	HL Cell Line HDLM-2
GSM311194	HL Cell Line KM-H2
GSM499723	HL Cell Line KM-H2
GSM499731	HL Cell Line KM-H2
GSM637959	HL Cell Line KM-H2
GSM499722	HL Cell Line L-1236
GSM499730	HL Cell Line L-1236
GSM637962	HL Cell Line L-1236

GSM311200	HL Cell Line L-428
GSM499721	HL Cell Line L-428
GSM499729	HL Cell Line L-428
GSM637960	HL Cell Line L-428
GSM499725	HL Cell Line L-540
GSM499726	HL Cell Line L-540
GSM637961	HL Cell Line L-540
GSM176298	HUVEC Cell Line
GSM176299	HUVEC Cell Line
GSM80691	Hypothalamus
GSM80692	Hypothalamus
GSM176268	Joint Tissue Synovium
GSM176269	Joint Tissue Synovium
GSM80687	Kdney Cortex
GSM80688	Kdney Cortex
GSM80732	Kidney Medulla
GSM80733	Kidney Medulla
GSM80729	Liver
GSM80730	Liver
GSM80707	Lung
GSM80712	Lung
GSM80736	Lymph Nodes
GSM80737	Lymph Nodes
GSM176231	Mammary Gland
GSM176232	Mammary Gland
GSM80709	Medulla
GSM80711	Medulla
GSM175901	Midbrain
GSM175903	Midbrain
GSM80718	Myometrium
GSM80719	Myometrium
GSM175838	Nipple Cross Section
GSM175840	Nipple Cross Section
GSM80769	Nodose Nucleus
GSM80770	Nodose Nucleus
GSM312937	Normal B Cells (Centroblasts)
GSM312938	Normal B Cells (Centroblasts)
GSM312939	Normal B Cells (Centroblasts)
GSM312940	Normal B Cells (Centroblasts)
GSM312941	Normal B Cells (Centroblasts)

GSM312887	Normal B Cells (Centrocytes)
GSM312890	Normal B Cells (Centrocytes)
GSM312893	Normal B Cells (Centrocytes)
GSM312894	Normal B Cells (Centrocytes)
GSM312895	Normal B Cells (Centrocytes)
GSM312877	Normal B Cells (Memory B Cells)
GSM312879	Normal B Cells (Memory B Cells)
GSM312882	Normal B Cells (Memory B Cells)
GSM312883	Normal B Cells (Memory B Cells)
GSM312886	Normal B Cells (Memory B Cells)
GSM312870	Normal B Cells (Naive B Cells)
GSM312872	Normal B Cells (Naive B Cells)
GSM312874	Normal B Cells (Naive B Cells)
GSM312875	Normal B Cells (Naive B Cells)
GSM312876	Normal B Cells (Naive B Cells)
GSM312942	Normal B Cells (Plasma Cells)
GSM312943	Normal B Cells (Plasma Cells)
GSM312944	Normal B Cells (Plasma Cells)
GSM312945	Normal B Cells (Plasma Cells)
GSM312946	Normal B Cells (Plasma Cells)
GSM80773	Occipital Lobe
GSM80774	Occipital Lobe
GSM80777	Oral Mucosa
GSM80778	Oral Mucosa
GSM175789	Ovary
GSM176131	Ovary
GSM175862	Parietal Lobe
GSM175864	Parietal Lobe
GSM176270	Penis
GSM176271	Penis
GSM80748	Pharyngeal Mucosa
GSM80749	Pharyngeal Mucosa
GSM80817	Pituitary Gland
GSM80818	Pituitary Gland
GSM175923	Prostate
GSM175924	Prostate
GSM175846	Putamen
GSM176020	Putamen
GSM80821	Salivary Gland
GSM80822	Salivary Gland

GSM175879	Saphenous Vein
GSM175880	Saphenous Vein
GSM175882	Skeletal Muscle
GSM175883	Skeletal Muscle
GSM175993	Skin
GSM176267	Skin
GSM175982	Small Intestine Jejunum
GSM176265	Small Intestine Jejunum
GSM175865	Spinal Cord
GSM175867	Spinal Cord
GSM80825	Spleen
GSM80826	Spleen
GSM175822	Stomach Cardiac
GSM175823	Stomach Cardiac
GSM80810	Stomach Fundus
GSM80811	Stomach Fundus
GSM80814	Stomach Pyloric
GSM80815	Stomach Pyloric
GSM175871	Substantia Nigra
GSM175873	Substantia Nigra
GSM176393	Substantia Nigra Pars Compacta
GSM176401	Substantia Nigra Pars Compacta
GSM176395	Substantia Nigra Reticulata
GSM176402	Substantia Nigra Reticulata
GSM175869	Subthalamic Nucleus
GSM175870	Subthalamic Nucleus
GSM175810	Synovial Membrane
GSM175811	Synovial Membrane
GSM175874	Temporal Lobe
GSM175876	Temporal Lobe
GSM176113	Testis
GSM176275	Testis
GSM176276	Testis
GSM176422	Testis
GSM176423	Testis
GSM175885	Thalamus
GSM175887	Thalamus
GSM176452	Thalamus Lateral Nuclei
GSM176454	Thalamus Lateral Nuclei
GSM176451	Thalamus Subthalamic Nucleus

GSM176453	Thalamus Subthalamic Nucleus
GSM175973	Thymus Gland
GSM176262	Thymus Gland
GSM80865	Thyroid Gland
GSM80866	Thyroid Gland
GSM175900	Tongue Main Corpus
GSM176014	Tongue Main Corpus
GSM175896	Tongue Superior With Papillae
GSM175898	Tongue Superior With Papillae
GSM80886	Tonsil
GSM80889	Tonsil
GSM175980	Trachea
GSM175981	Trachea
GSM175889	Trigeminal Ganglia
GSM175891	Trigeminal Ganglia
GSM80911	Urethra
GSM80912	Urethra
GSM175878	Vagina
GSM176129	Vagina
GSM175829	Ventral Tegmental Area
GSM175831	Ventral Tegmental Area
GSM175893	Vestibular Nuclei Superior
GSM175894	Vestibular Nuclei Superior
GSM80897	Vulva
GSM80898	Vulva
