# Supplementary figures and images for: Characterization of Alstrom Syndrome 1 (ALMS1) Transcript Variants in Hodgkin Lymphoma Cells
Source: PLoS One. 2017 Jan 30;12(1):e0170694. doi: 10.1371/journal.pone.0170694 (PMC5279758; doi:10.1371/journal.pone.0170694)

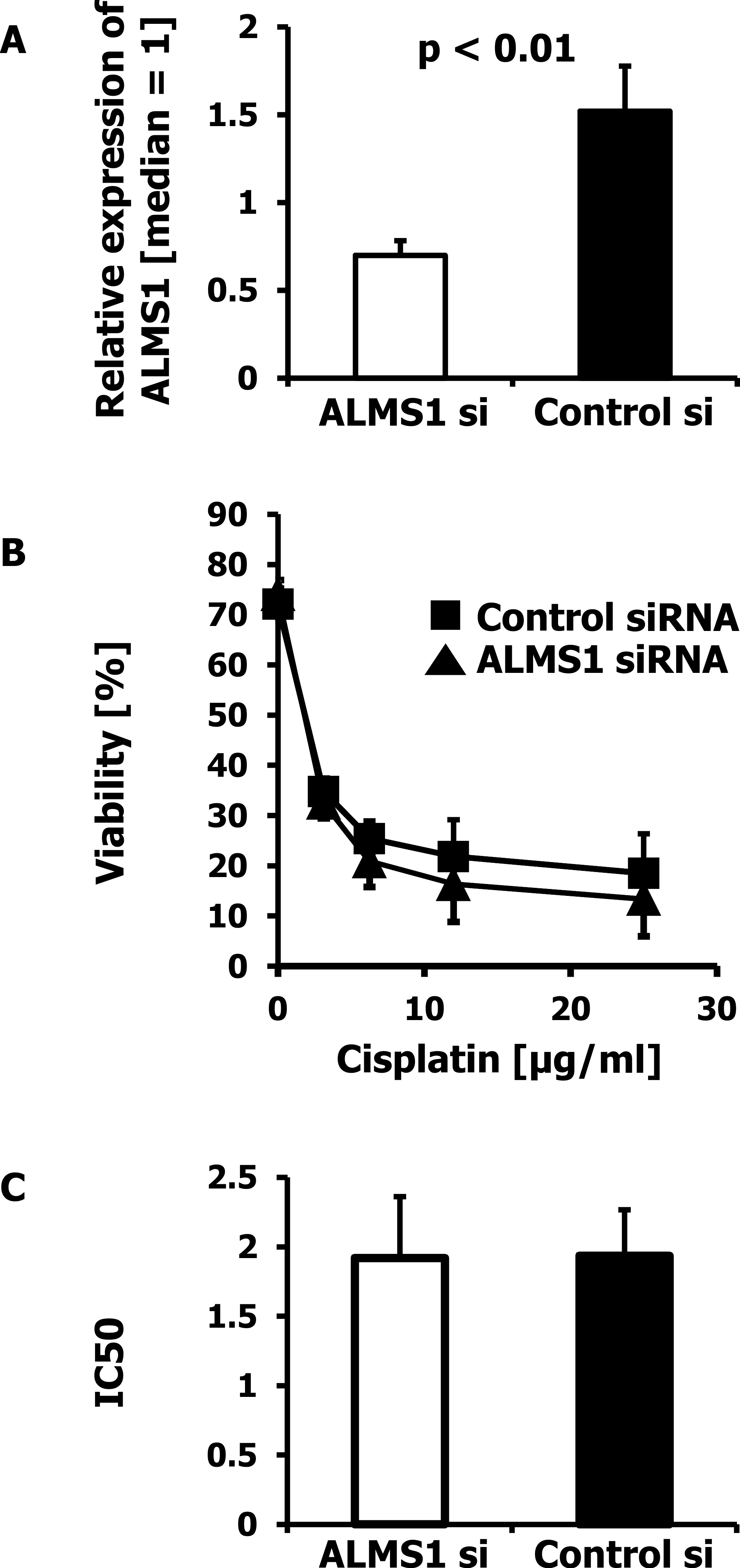

Supplement: S1 Fig — ALMS1 specific siRNA was used for knock-down of ALMS1 in HL cell line KM-H2. Presented are means and standard deviations from 3 independent transfection experiments (n = 4, control si RNA; n = 6, ALMS1 siRNA) A) ALMS1 knock-down was validated by qRT-PCR using primer combination 28. The median of all samples was set as one and HPRT1 (primer combination 39) was used as housekeeping control. Statistical significance was tested by Student’s t test. B) Cisplatin sensitivity was analyzed after transfection of KM-H2 cells with siRNAs. Presented are percentages of viable propidium iodide negative cells. C) 50%-inhibitory concentrations (IC50) for cisplatin treated KM-H2 cells after transfection were calculated by using the IC50 Tool Kit (http://www.ic50.tk/). (TIF) [file pone.0170694.s002.tif]

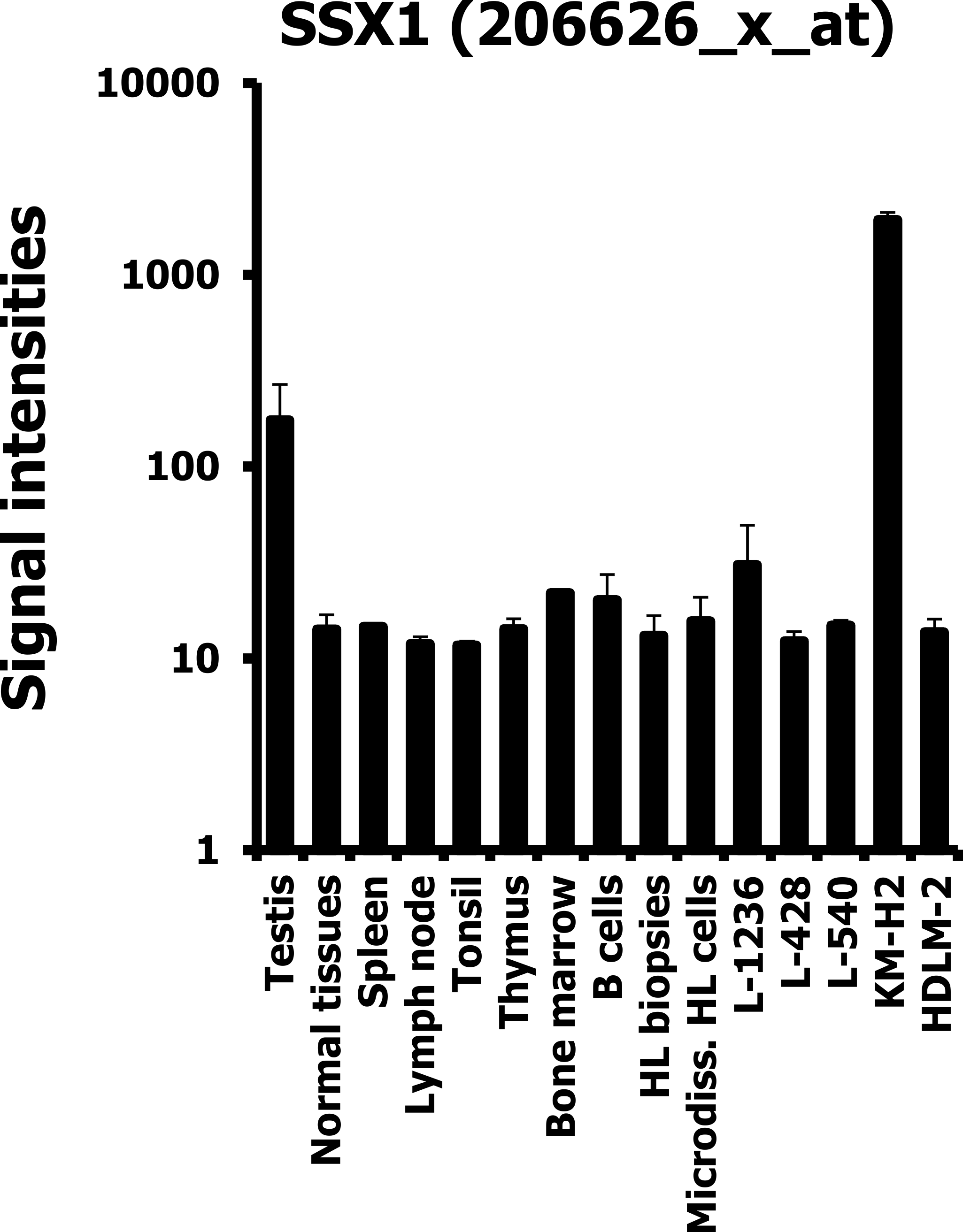

Supplement: S2 Fig — Presented are signal intensities from the microarray data sets summarized in S1 Table. Cel files were downloaded from the Gene Expression Omnibus data base and analyzed with Affymetrix Expression Console using Robust Multi-Array Analysis (RMA) algorithm. Presented are signal intensities from probe set 206626_x_at that is specific for synovial sarcoma X family member 1 (SSX1). Microdiss. HL cells: = HL cells that have been micro-dissected from HL biopsies. (TIF) [file pone.0170694.s003.tif]

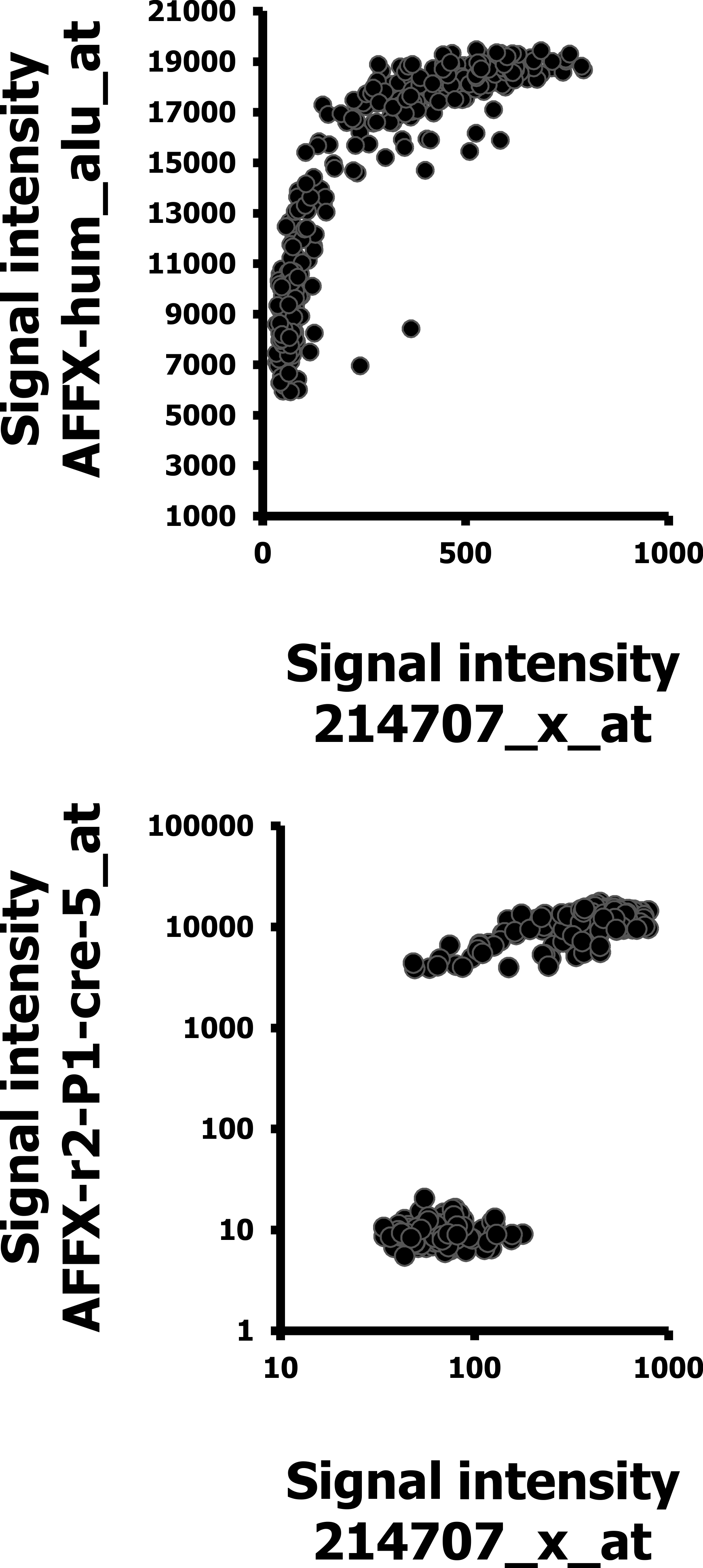

Supplement: S3 Fig — Presented are signal intensities from the microarray data sets summarized in S1 Table. Cel files were downloaded from the Gene expression Omnibus data base and analyzed with Affymetrix Expression Console using Robust Multi-Array Analysis (RMA) algorithm. Presented are signal intensities from probe set 214707_x_at in correlation to the indicated control probe sets. (TIF) [file pone.0170694.s004.tif]
